# Integrative multi-omics identifies PANX2 as a ferroptosis suppressor and potential therapeutic target in clear cell renal cell carcinoma

**DOI:** 10.3389/fimmu.2026.1883667

**Published:** 2026-08-03

**Authors:** Xinglin Li, Yiqi Xiong, Jiyin Wang, Wenying Qiao, Liqian Yu, Jitao Wu, Yonghong Zhang, Ronghua Jin, Dongxu Zhang

**Affiliations:** 1 Department of Urology, The Affiliated Hospital of Qingdao University, Qingdao, China; 2 Medical College, Qingdao University, Qingdao, China; 3 Beijing Youan Hospital, Capital Medical University, Beijing, China; 4 Beijing Ditan Hospital, Capital Medical University, Beijing, China; 5 Department of Urology, The Affiliated Yantai Yuhuangding Hospital of Qingdao University, Yantai, China

**Keywords:** clear cell renal cell carcinoma, ferroptosis, PANX2, single-cell RNA sequencing, spatial transcriptomics, tumor immunity

## Abstract

**Background:**

Clear cell renal cell carcinoma (ccRCC) is metabolically primed for ferroptosis, yet the prognostic relevance and mechanistic contribution of ferroptosis-related genes remain incompletely defined. This study aimed to identify ferroptosis-associated biomarkers with prognostic value and to clarify their functional relevance in ccRCC progression.

**Methods:**

We integrated single-cell RNA sequencing, bulk RNA-seq, spatial transcriptomics, and machine-learning-based feature selection to identify ferroptosis-related prognostic genes in ccRCC. A four-gene risk model and an integrated nomogram were constructed and evaluated in independent cohorts. PANX2 was prioritized for experimental validation using stable knockdown models, RNA sequencing, lipid peroxidation and iron probes, redox assays, Western blot, luciferase reporter assays, xenografts, and an immunocompetent murine renal carcinoma model.

**Results:**

A four-gene prognostic signature (CA9, PVT1, RRM2, and PANX2) was identified and used to construct a risk model with consistent predictive performance in the training and external validation cohorts. Among these genes, PANX2 was predominantly enriched in epithelial tumor compartments and had not been functionally characterized in ccRCC. PANX2 knockdown inhibited ccRCC cell proliferation, reduced antioxidant capacity, increased intracellular Fe^2+^ accumulation and lipid peroxidation, and sensitized cells to erastin-induced ferroptotic death. Mechanistically, PANX2 loss was associated with reduced Akt/mTOR pathway activity and diminished SLC7A11 expression; rescue with an Akt activator or SLC7A11 overexpression attenuated ferroptosis-associated phenotypes. In an immunocompetent murine renal carcinoma model, PANX2 knockdown was accompanied by increased infiltration of CD45+ leukocytes, CD3+ T cells, and CD8+ T cells, supporting a potential link between PANX2-dependent ferroptosis resistance and the tumor immune contexture.

**Conclusions:**

This study identifies PANX2 as a ccRCC-relevant suppressor of ferroptosis and supports the involvement of a PANX2-Akt/mTOR-SLC7A11-associated signaling axis in redox homeostasis and tumor progression. The ferroptosis-related prognostic model and nomogram may support risk stratification, while PANX2 represents a candidate therapeutic vulnerability that warrants further mechanistic and translational validation.

## Introduction

Renal cell carcinoma (RCC) is the predominant malignant tumor of the kidney, with clear cell RCC (ccRCC) representing the most common histological subtype (1–4). Although surgery, tyrosine kinase inhibitors, immune checkpoint inhibitors, and combination regimens have improved clinical management, recurrence and treatment resistance remain major obstacles, particularly in advanced disease (2, 3). Current prognostic assessment still relies largely on clinicopathological parameters, whereas recent studies have highlighted non-invasive imaging and inflammatory indices, such as computed tomography-derived perinephric adipose tissue features and the C-reactive protein-to-albumin ratio, as complementary tools for risk evaluation in non-metastatic ccRCC (5). These developments underscore the need for integrated prognostic frameworks that connect clinical risk stratification with biologically interpretable molecular mechanisms rather than relying on purely statistical signatures.

Ferroptosis, a regulated cell death (RCD) pathway first defined by Stockwell et al. approximately ten years ago (6), is characterized by iron-dependent accumulation of lethal lipid peroxides. This mechanism distinguishes it from apoptosis and other RCD modalities (7). Malignant cells typically exhibit elevated iron pools and reactive oxygen species (ROS) that sustain their metabolic demands and proliferative capacity (8). Consequently, inducing ferroptosis may represent a promising therapeutic strategy for cancers resistant to standard chemotherapeutic or molecular-targeted agents. However, the ferroptosis-related genes that determine prognosis, therapy response, and immune contexture in ccRCC remain insufficiently characterized.

Single-cell RNA sequencing (scRNA-seq) enables high-resolution profiling of heterogeneous tumor ecosystems, whereas spatial transcriptomics preserves tissue architecture and supports spatial interpretation of tumor and stromal compartments (9–11). When combined with bulk transcriptomic cohorts and transparent feature-selection strategies, these technologies can help identify prognostic biomarkers that are both statistically reproducible and biologically interpretable. Recent studies have increasingly demonstrated that integrating machine learning with molecular profiling provides an effective framework for disease characterization and biomarker discovery across diverse clinical settings (12–14).

In this study, we integrated scRNA-seq, bulk RNA-seq, spatial transcriptomics, and machine learning to identify ferroptosis-related prognostic genes in ccRCC. We developed a four-gene prognostic signature and nomogram, then prioritized PANX2 for experimental validation because its role in ccRCC remained unclear despite previous reports implicating PANX2 in other malignancies. We show that PANX2 depletion disrupts redox homeostasis, increases lipid peroxidation and intracellular iron accumulation, and sensitizes ccRCC cells to ferroptosis. We further provide evidence that PANX2 is associated with Akt/mTOR-SLC7A11 signaling and may influence the tumor immune contexture, while explicitly acknowledging the mechanistic and model-related boundaries of these conclusions.

## Methods

This study was reported in accordance with the REMARK criteria (15).

### Transcriptome data download and processing

The Cancer Genome Atlas (TCGA) dataset for ccRCC was downloaded from UCSC Xena (https://portal.gdc.cancer.gov/). The validation dataset (N = 73) was obtained from the International Cancer Genome Consortium (ICGC) data portal (https://dcc.icgc.org/), the E-MTAB-1980 cohort from ArrayExpress, and the GSE167573 dataset from the Gene Expression Omnibus (GEO). All FPKM (fragments per million mapped reads per thousand base pairs) data were log2-transformed before downstream analysis.

### Single-cell dataset processing and analysis

The GSE159115 dataset, including eight tumor tissue samples and six normal tissue samples, was downloaded from the Gene Expression Omnibus (GEO). The scRNA-seq expression matrix was processed using the R package Seurat. Quality control (QC) was performed by requiring each gene to be expressed in at least three cells and each cell to express at least 250 genes. Mitochondrial and ribosomal RNA percentages were calculated using the PercentageFeatureSet function. Cells with more than 200 detected genes, more than 500 UMI counts, and less than 25% mitochondrial gene content were retained. The NormalizeData function was used to normalize gene expression data, and FindVariableFeatures was used to identify 2,000 highly variable genes (HVGs). Principal component analysis (PCA) was then performed for dimensionality reduction using the highly variable genes. The top 20 principal components were used for clustering analysis and for UMAP visualization based on the elbow plot (**Supplementary Figure 1H**). Next, the FindNeighbors and FindClusters functions were applied to cluster cells on a shared-nearest-neighbor graph. This workflow was selected because graph-based clustering methods implemented in Seurat have shown robust performance across diverse scRNA-seq datasets, while previous benchmarking studies have demonstrated that clustering algorithm selection and handling of dropout-related sparsity can substantially influence downstream cell-type identification and biological interpretation (16). We evaluated resolutions ranging from 0.1 to 1.0 using clustree-based cluster stability analysis, and resolution = 0.4 was selected (**Supplementary Figure 1I**). To assess and correct potential batch effects across samples, PCA embeddings before integration were first used to compute a pre-integration UMAP embedding, enabling visualization of sample-driven clustering before correction. Batch correction was then performed using the Harmony algorithm (RunHarmony), with sample identity (orig.ident) specified as the batch variable. Comparative PCA and UMAP plots before and after Harmony integration confirmed that sample-associated batch effects were substantially reduced (**Supplementary Figures 1A–H**). After initial QC filtering, potential doublets were identified using the DoubletFinder package. Briefly, artificial doublets were generated with pN = 0.25, and the optimal pK value (pK = 0.09) was selected using the paramSweep/BCmetric strategy. The expected number of doublets (nExp) was estimated based on the cell loading rate of the 10x Genomics platform. Cells classified as doublets (DF.classification = “Doublet”) were removed.

For differential expression analysis across clusters or subclusters, the FindAllMarkers function in Seurat was used, with a log-fold change threshold greater than 0.25, a P value less than 0.05, and an expression ratio greater than 0.1. Cell clusters were subsequently annotated based on marker genes from the CellMarker database and genes reported in the literature. Gene-set scores were calculated using the AddModuleScore function.

### Spatial transcriptomics data processing and analysis

Spatial transcriptomics data for ccRCC were obtained from GSE250163. Visium spatial gene expression data, FASTQ files, and feature-barcode (FB) matrices were generated using 10x Genomics Space Ranger v1.2.2. The FB matrix was loaded into Seurat v4.3.1 for subsequent analysis. The SCTransform function in Seurat was used to standardize the spatial transcriptomics data, whereas RunPCA and RunUMAP were used for dimensionality reduction and clustering. To analyze the spatial localization of single-cell clusters in ccRCC, we applied robust cell-type decomposition (RCTD; spacexr package), using the integrated scRNA-seq dataset as a reference to decompose the cellular composition of spatial spots. RCTD was run with doublet_mode = “doublet”, which was selected as the optimal setting after comparison with the “full” mode, and the resulting mixing weights were added to the Seurat object metadata.

Clustering and spatial enhancement were performed using BayesSpace. Spatial clustering was conducted with q = 5 under the “Visium” platform setting, and enhanced-resolution clustering was performed with q = 6 to refine spot-level boundaries. Target-gene expression of target genes was visualized using FeaturePlot, and marker-gene expression was further refined using BayesSpace feature enhancement (enhanceFeatures). Although alternative computational approaches, such as MLSpatial, have been proposed to reconstruct spatial cell distributions by learning spatial features from scRNA-seq data, we selected RCTD for reference-based spot deconvolution because the present study used Visium data with available spatial coordinates and aimed to estimate cell-type composition within spatial spots rather than to reconstruct cell positions from scRNA-seq data alone (17).

### Differential gene expression analysis

DEGs between tumor and adjacent normal tissues in the scRNA-seq dataset were identified using the FindMarkers function in Seurat. For bulk RNA-seq differential expression analysis, DESeq2 was applied with default parameters using raw count matrices as input to identify DEGs between tumor and adjacent normal tissues. In addition, samples were classified into high-risk and low-risk groups according to the median risk score for subsequent differential analysis. Multiple-testing correction was performed using the Benjamini-Hochberg method, and pathways with FDR < 0.05 were considered statistically significant.

### Identification of ferroptosis-related prognostic genes

Ferroptosis-related genes were extracted from the GeneCards database and high-quality peer-reviewed literature for downstream analysis. Intersecting the DEGs identified between tumor and normal tissues in both the scRNA-seq dataset and TCGA cohort with ferroptosis-related genes yielded 23 candidate genes. Multiple machine-learning algorithms were applied to identify robust prognostic genes, including LASSO regression, SVM-RFE, Gradient Boosting Machine (GBM), and Random Forest. GBM and Random Forest ranked genes based on feature importance, with genes showing importance scores > 1 retained for further analysis. SVM-RFE selected the optimal feature subset according to the minimum cross-validation error, whereas LASSO regression retained genes with non-zero coefficients at the optimal lambda determined by 10-fold cross-validation. The intersection of genes selected across multiple algorithms (LASSO, SVM-RFE, Random Forest, and GBM) resulted in 10 key genes for further analysis.

### Construction of ferroptosis-related prognostic signatures

Univariate and multivariate Cox regression analyses were used to screen genes associated with prognosis in ccRCC patients, from which a risk score was developed using the selected genes. Based on the risk score, patients were stratified into high- and low-ferroptosis-risk groups. Kaplan-Meier survival analysis with the log-rank test was performed to evaluate survival differences between the two groups. Time-dependent receiver operating characteristic (ROC) curves were generated to assess the predictive ability of the prognostic signature. To obtain more stable estimates of model performance, we further calculated the concordance index (C-index), calibration, and time-dependent AUC values together with their 95% confidence intervals using 1,000 bootstrap resamples.

### Enrichment analysis

Enrichment analysis was performed using DEGs between the high-risk and low-risk groups in the TCGA cohort. ClusterProfiler was used to perform Gene Ontology (GO), Kyoto Encyclopedia of Genes and Genomes (KEGG), and gene set variation analysis (GSVA) to enrich the cell populations. GSVA is a non-parametric and unsupervised method primarily used to evaluate pathway enrichment across samples. For GSVA, the GO_BP, GO_MF, GO_CC, KEGG, HALLMARK, REACTOME, and WikiPathways gene sets from the MSigDB database (https://www.gsea-msigdb.org/gsea/msigdb) were used to assess pathway enrichment among different samples.

### Immune infiltration

The ESTIMATE algorithm was used to estimate stromal and immune cell content in tumor tissues. We compared the immune score, stromal score, and ESTIMATE score between the high-risk and low-risk groups using the estimate package. Furthermore, we evaluated 28 immune-infiltrating cell components using single-sample gene set enrichment analysis (ssGSEA), including activated CD4+ T cells, activated dendritic cells, central memory CD4+ T cells, immature B cells, type 2 helper T cells, activated B cells, activated CD8+ T cells, regulatory T cells, type 1 helper T cells, neutrophils, effector memory CD4+ T cells, effector memory CD8+ T cells, T follicular helper cells, NKT cells, CD56bright NK cells, immature dendritic cells, monocytes, plasmacytoid dendritic cells, NK cells, effector memory T cells, CD56dim NK cells, gamma delta T cells, memory B cells, mast cells, and eosinophils. To further validate the robustness of immune infiltration patterns inferred from ssGSEA, we performed cell-type deconvolution using the xCell algorithm. The TPM matrix was uploaded into the immunedeconv framework, and the xCell method was applied to estimate enrichment scores for 39 immune and stromal cell populations.

### Tumor immune dysfunction and exclusion and immune checkpoint-related gene expression

To predict the response to immune checkpoint blockade, we used the TIDE method developed by Jiang et al. (http://tide.dfci.harvard.edu/), hich models mechanisms of tumor immune evasion, including T-cell dysfunction and T-cell exclusion. In our study, FPKM expression values were normalized toward the all-sample mean according to the official TIDE preprocessing protocol before submission to the TIDE platform. To further identify the ccRCC patients who may derive greater benefit from immune checkpoint inhibitor (ICI) therapy, we compared the expression levels of immune checkpoint-related genes expression between different groups.

### Drug sensitivity analysis

Drug sensitivity was predicted using the FPKM-normalized expression matrix, which was log2-transformed before being mapped to the GDSC reference datasets. Drug sensitivity in ccRCC patients was estimated using the oncoPredict package and expression data from the TCGA-KIRC cohort.t.

### Nomogram

A nomogram was established by combining the prognostic signature with clinical characteristics to predict the prognosis in patients with ccRCC. The accuracy and reliability of the nomogram were assessed using 3- and 5-year ROC curves, calibration plots, and decision curve analysis (DCA).

### Cell culture

Human ccRCC cell lines (786-O and Caki-1) were obtained from the Chinese Academy of Sciences Cell Bank (Shanghai, China) and were cultured in RPMI-1640 medium supplemented with 10% fetal bovine serum (FBS) and 1% penicillin-streptomycin. Cultures were maintained at 37°C in a humidified 5% CO_2_ atmosphere, with periodic testing for mycoplasma contamination.

### shRNA and stable cell lines

Lentiviral transfer plasmids included PLKO.1-puro (control) and two PANX2-targeting constructs: PLKO.1-shPANX2-1-puro, and PLKO.1-shPANX2-2-puro. Lentiviruses were packaged in HEK293T cells using co-transfection with PSPAX2 and PMD2.G packaging plasmids. Viral supernatants were subsequently used to transduce 786-O and Caki-1 cells. Transduced cells underwent selection in puromycin/blasticidin-containing medium for ≥72 h to generate stable polyclonal lines. The target sequences of PANX2 shRNA are provided in **Supplementary Table 1**.

### Real-time reverse transcription quantitative PCR

Total RNA was extracted from tissue samples using the Total RNA Extraction Kit (Dakewe Biotech, China). RNA concentration and purity were determined using a NanoDrop One spectrophotometer (Thermo Fisher Scientific, USA). Reverse transcription was performed with 1 μg RNA using the TaKaRa RT-PCR Kit (Takara Bio, Japan). Quantitative PCR amplification was performed with ChamQ SYBR qPCR Master Mix (Vazyme Biotech, China). Primer sequences are listed in **Supplementary Table 2**.

### Western blot

Proteins were extracted from cells using RIPA lysis buffer containing protease and phosphatase inhibitors. Protein concentrations were determined with a BCA assay kit (Thermo Fisher Scientific, USA). Lysates (60 μg) underwent separation via SDS-PAGE and electrophoretic transfer onto PVDF membranes. After blocking with 5% skim milk, membranes were incubated overnight at 4°C with primary antibodies, followed by horseradish peroxidase-conjugated secondary antibodies. Protein bands were visualized using enhanced chemiluminescence substrate (Merck, Germany). Antibody specifications are provided in **Supplementary Table 3**.

### Cell counting kit-8 assay

Cell proliferation was determined using Cell Counting Kit-8 (CCK-8; MedChemExpress, China). Cells (5×10³/well) were plated in 96-well plates and subjected to specified treatments. Following treatment, 10 μl CCK-8 reagent was added to 100 μl fresh culture medium per well, then incubated for 2 h at 37 °C in a humidified 5% CO_2_ atmosphere. Absorbance at 450 nm was measured using a microplate reader and normalized against blank and vehicle (DMSO) controls. The proliferation ability of cells was quantified through daily absorbance measurements over a 72-h period.

### 5-Ethynyl-2’-deoxyuridine assay

EdU incorporation assays were performed using a commercial EdU detection kit (Beyotime, China) according to the manufacturer’s instructions. Proliferating cells were detected and quantified by flow cytometric analysis.

### Animal studies

Six-week-old male BALB/c nude mice and female immunocompetent BALB/c mice were maintained under specific pathogen-free conditions (no more than five mice per cage) after one week of acclimatization. Mice were randomly assigned to control and experimental groups using a computer-generated sequence. To assess tumor growth, 786-O cells (1 x 10^6 cells in 100 μL PBS) were injected subcutaneously into the shaved dorsal flank of nude mice (n = 5 per group). To explore immune microenvironmental changes in an immunocompetent renal carcinoma setting, Renca cells were injected into BALB/c mice under identical conditions (n = 6 per group). These two models were used for distinct purposes and were not considered directly interchangeable: the 786-O xenograft model assessed human ccRCC tumor growth, whereas the Renca/BALB/c model provided a murine system for evaluating tumor-infiltrating immune cells. Palpable tumors typically developed within 5–7 days. Tumor growth was monitored every three days by an investigator blinded to group allocation. When endpoint criteria were reached (tumor diameter >= 2 cm, ulceration, or >10% body weight loss), mice were anesthetized by intraperitoneal sodium pentobarbital (50 mg/kg) and euthanized by cervical dislocation. Tumors were then excised. Sample size was determined based on pilot data. Mice that failed to develop tumors were excluded from analysis, and all tumor-bearing animals meeting endpoint criteria were included without exclusion of outliers. All procedures complied with ARRIVE guidelines (18) and were approved by the Institutional Animal Ethics Committee.

### RNA sequences

Total RNA was isolated from stable PANX2-knockdown and control cell models (biological triplicates) using TRIzol reagent. RNA-seq libraries were constructed and sequenced by Gene+ (Beijing, China). Raw sequencing reads were quality-assessed with FastQC, followed by differential expression analysis using DESeq2.

### Measurement of NADPH/NADP^+^ and GSH/GSSG ratios

Intracellular NADPH/NADP^+^ and GSH/GSSG ratios were measured using the respective commercially available assay kits (Beyotime, China) according to the manufacturer’s instructions.

### Lipid peroxidation assay

Lipid peroxidation was evaluated using BODIPY 581/591 C11 (2-10 μM; MedChemExpress, USA) and Liperfluo (1 μM; Dojindo, Japan) probes. Following manufacturer-specified protocols, cells were stained in serum-free medium and analyzed by flow cytometry.

### Measurement of intracellular Fe^2+^

Intracellular Fe^2+^ was detected and quantified using the FerroOrange fluorescent probe (Dojindo, Japan) according to the manufacturer’s protocol, followed by flow cytometric analysis.

### Immunohistochemical analysis

IHC staining was performed on formalin-fixed, paraffin-embedded sections from the patient tissue microarray and xenograft tumors. After deparaffinization, antigen retrieval, and blocking, sections were incubated with primary antibodies overnight at 4°C. Detection was performed using horseradish peroxidase-conjugated secondary antibodies and DAB substrate, followed by hematoxylin counterstaining. Clinical tissue microarray procedures complied with the Declaration of Helsinki, and written informed consent was obtained from all participants before tissue collection. Stained slides were digitized and independently evaluated by two pathologists blinded to clinical and experimental information. Staining intensity was semi-quantitatively evaluated using the H-score system.

### Dual-luciferase reporter gene assay

Plasmids were introduced into cells at approximately 70% confluence using Lipofectamine 2000 transfection reagent. Each well received 300 ng of the experimental plasmid and 10 ng of a Renilla luciferase control plasmid. Luciferase activity was assessed 36 hours after transfection using a dual-luciferase reporter assay system (Promega), with firefly luciferase values normalized to the corresponding Renilla luciferase readings. Primer sequences used for cloning the SLC7A11 promoter into the luciferase reporter vector are provided in **Supplementary Table 4**.

### Statistical analysis

All data were derived from a minimum of three independent biological replicates and presented as mean ± SD. Statistical analyses were performed using GraphPad Prism 8, with specific tests detailed in corresponding figure legends. Unless otherwise stated, all statistical tests were two-sided. P values were adjusted for multiple testing using the Benjamini-Hochberg method false discovery rate (FDR) method. Significance levels were denoted as follows: *p < 0.05, **p < 0.01, ***p < 0.001; ns indicates non-significance (p ≥ 0.05).

## Results

### Identification of ferroptosis-related genes in ccRCC patients

**Figure 1:**
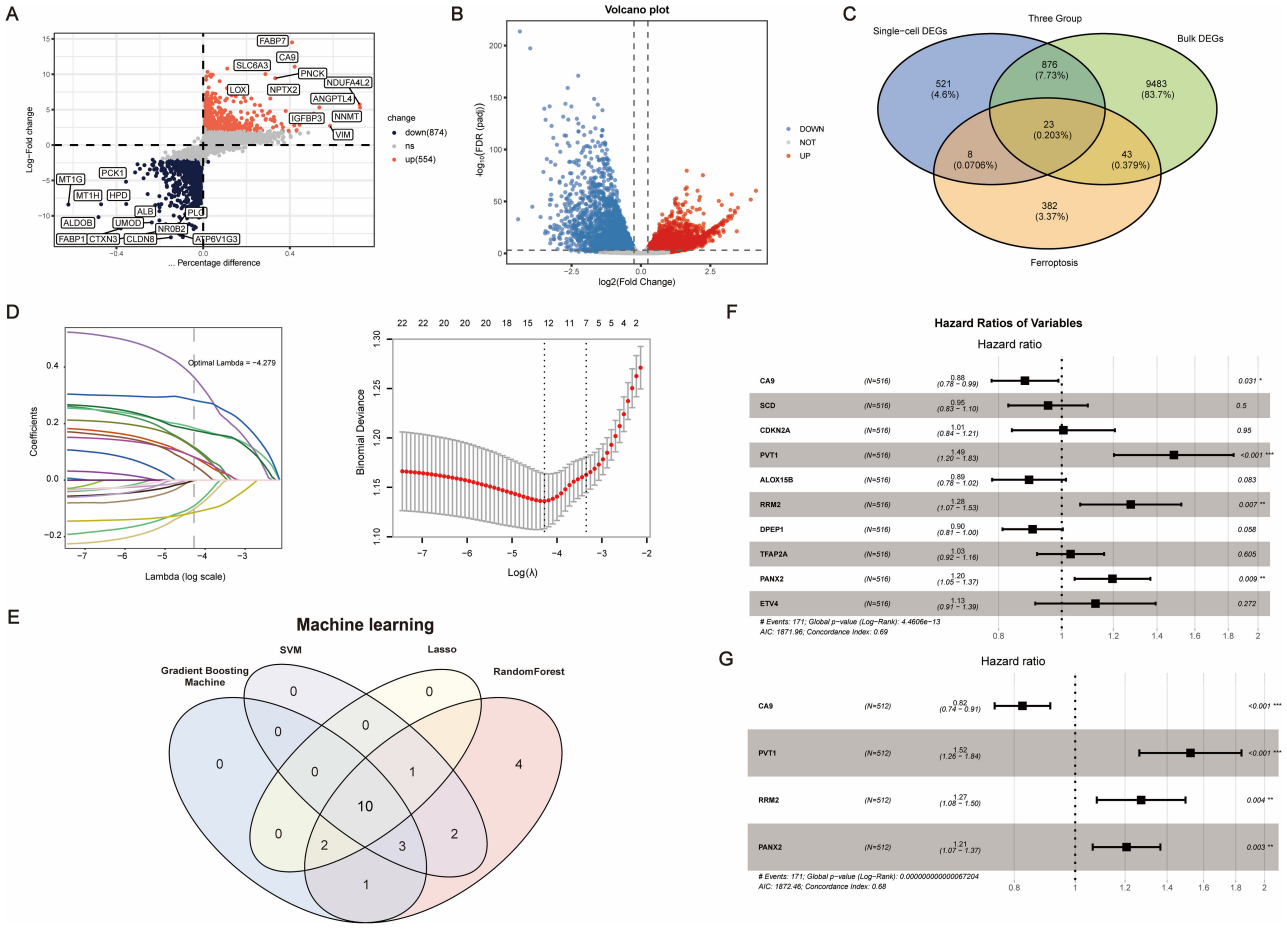
Identification of ferroptosis-related prognostic genes in ccRCC through integrated bioinformatic analysis. **(A)** Volcano plot displaying DEGs between tumor and normal tissues in the GSE159115 single-cell RNA-seq dataset. DEGs were identified using the Wilcoxon rank-sum test with Benjamini-Hochberg FDR correction. **(B)** Volcano plot of DEGs from the TCGA-KIRC cohort. DEGs were analyzed using DESeq2 with Wald tests and BH FDR correction. **(C)** Venn diagram showing the intersection of DEGs from GSE159115, TCGA-KIRC, and a ferroptosis-related gene set from Genecards. **(D, E)** Feature selection plots from machine learning algorithms (LASSO, SVM, GBM, Random Forest) applied to identify hub genes. **(F)** Forest plot of multivariate Cox regression analysis for prognostic gene selection. **(G)** Multivariate Cox regression results of the four ferroptosis-related genes significantly associated with ccRCC prognosis. Hazard ratios and 95% confidence intervals were computed, and significance was assessed using two-sided Wald tests. **p < 0.01, ***p < 0.001.

To identify key ferroptosis-related genes in ccRCC, we conducted differential gene expression analysis of tumor and adjacent normal tissues from the single-cell dataset GSE159115 and the TCGA-KIRC cohort (adjusted P value < 0.05 and |log_2_FC| > 2), identifying 634 (**Figure 1A**) and 8,116 DEGs (**Figure 1B**), respectively. A ferroptosis-related gene set comprising 406 genes was obtained from GeneCards. The intersection of these three gene sets yielded 23 candidate genes (**Figure 1C**), which were subsequently analyzed using machine-learning algorithms, including LASSO, SVM-RFE, GBM, and Random Forest, to identify hub signatures (**Supplementary Figures 2A–C**; **Figures 1D, E**). Then, univariate and multivariate Cox regression were performed to give a further analysis. Genes with univariate analysis P value <0.05 were used for multivariate analysis (**Figure 1F**), and four ferroptosis-related prognostic genes were identified, encompassing CA9 (HR: 0.82, 95% CI: 0.74-0.91, P <0.001), PVT1 (HR: 1.52, 95% CI: 1.26-1.84, P <0.001), RRM2 (HR: 1.27, 95% CI: 1.08-1.50, P = 0.004), and PANX2 (HR:1.21, 95%CI: 1.07-1.37, P = 0.003) (**Figure 1G**). We further verified that all four ferroptosis-related prognostic genes were recorded in the FerrDb database as experimentally supported ferroptosis regulators (**Supplementary Table 5**).

### Development of a prognostic signature of ccRCC

**Figure 2:**
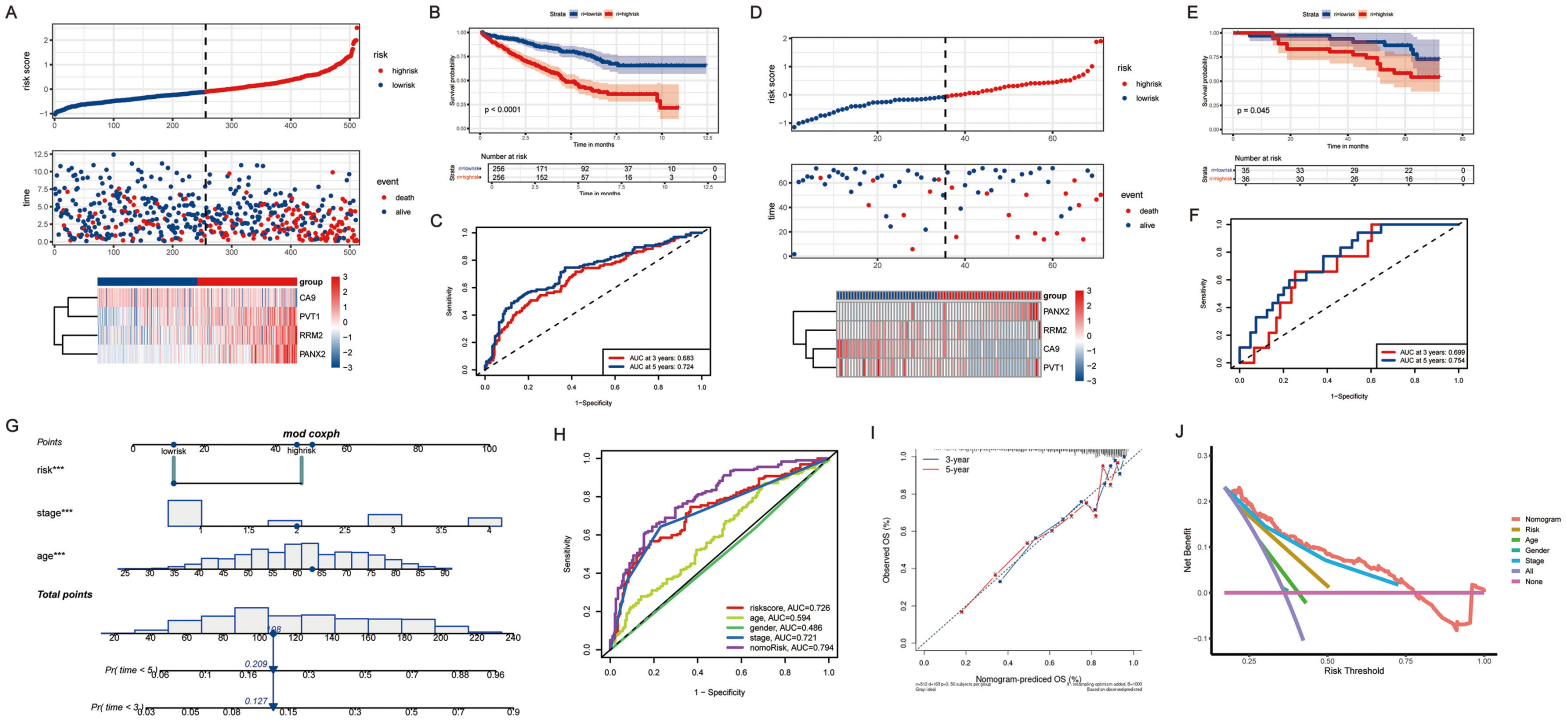
Development and validation of a ferroptosis-related prognostic model and clinical nomogram in ccRCC. **(A)** Schematic representation of the prognostic risk scoring model based on four ferroptosis-related genes. **(B)** Kaplan-Meier survival curves comparing high-risk and low-risk groups in the training cohort. Survival differences were evaluated using the two-sided log-rank test. **(C)** ROC curves evaluating the predictive accuracy of the model at 3 and 5 years in the training cohort. **(D)** Overview of the external validation cohort (ICGC) used for model assessment. **(E)** Kaplan-Meier survival analysis of high-risk and low-risk groups in the validation cohort. Survival differences were evaluated using the two-sided log-rank test. **(F)** ROC curves assessing model performance at 3 and 5 years in the external cohort. **(G)** Nomogram integrating the ferroptosis-related gene signature with clinical parameters for risk prediction. **(H)** ROC analysis comparing the predictive accuracy of the nomogram against individual prognostic factors. **(I)** Calibration curves depicting the agreement between nomogram-predicted and actual survival outcomes. **(J)** Decision curve analysis (DCA) comparing the nomogram, ferroptosis-related risk score, and clinicopathological variables for survival prediction in patients with ccRCC across different threshold probabilities

The prognostic risk-scoring model was established based on the four ferroptosis-related prognostic genes (**Figure 2A**). The model demonstrated moderate discriminative performance, with a C-index of 0.68 (95%CI: 0.634-0.724), and the 3- and 5-year AUCs of 0.683 (95%CI: 0.621-0.741) and 0.724 (95%CI: 0.665-0.778, **Figure 2C**). In the external ICGC cohort (**Figure 2D**), the 3- and 5-year AUCs of the model were 0.699 and 0.754, supporting the external validity of the model (**Figure 2F**). Consistently, the model demonstrated acceptable discriminative ability in two additional external validation datasets (**Supplementary Figures 3C, G**). In the E-MTAB-1980 cohort, the 3- and 5-year AUCs were 0.810 and 0.826, whereas in the GSE167573 cohort, the 3- and 5-year AUCs were 0.966 and 0.702, respectively (**Supplementary Figures 3E, I**). The relatively high 3-year AUC in the GSE167573 cohort may be partly related to the limited number of events. Moreover, calibration analyses at both 3 and 5 years showed good agreement between predicted and observed survival probabilities (**Supplementary Figures 3A, B, F, J**). To assess the incremental prognostic value of the signature, we compared the clinical model (age, sex, and stage) with the combined model incorporating the risk score. At 3 and 5 years, the combined model showed a significantly improved reclassification ability, with a continuous NRI values of 0.168 (95% CI: 0.004–0.291, P = 0.040) and 0.293 (95% CI: 0.078–0.413, P = 0.013). The IDI was 0.026 (95% CI: -0.006–0.067, P = 0.093) at 3 years, which did not reach statistical significance, and 0.05 (95% CI: 0.014–0.102, P = 0.007) at 5 years, indicating a significant improvement. The prognostic risk scores for ccRCC patients were calculated using the gene expression values and the regression coefficients of the model, and patients were subdivided into high-risk and low-risk groups on the basis of risk score. Risk score = (−0.190 × expression of CA9) + (0.415 × expression of PVT1) + (0.242 × expression of RRM2) + (0.193 × expression of PANX2). Survival analysis revealed that the prognosis of ccRCC patients in the high-risk group was worse than that in the low-risk group in both the training cohort and the validation cohort (**Figures 2B, E**). Consistently, stratified analyses across age, sex, and tumor stage subgroups showed the same trend, with high-risk patients exhibiting poorer survival in all subgroups, further supporting the robustness and clinical stability of the prognostic signature (**Supplementary Figure 3K**). The association between the four-gene risk score and clinicopathological characteristics was further evaluated. The high-risk group was positively associated with death and advanced tumor stage (**Supplementary Figure 4**). These results suggest that the ferroptosis-related risk score is closely linked to malignant progression and poor clinical outcome in ccRCC, indicating that the four-gene signature may capture both prognostic risk and clinically relevant tumor aggressiveness.

### Construction of a nomogram

To improve risk stratification in ccRCC patients, we constructed a nomogram that integrated ferroptosis-related gene signatures with clinical parameters, including age and tumor stage (**Figure 2G**). The nomogram demonstrated higher predictive accuracy compared to individual prognostic indicators, as evidenced by ROC curve analysis (**Figure 2H**). Calibration curves indicated strong agreement between predicted and observed survival outcomes (**Figure 2I**). DCA further confirmed the superior clinical utility of the nomogram over conventional clinical metrics (**Figure 2J**). From a practical perspective, this nomogram may provide individualized survival-risk estimates and help distinguish patients with similar clinicopathological features but different molecular risk profiles. Such risk stratification could support more tailored postoperative surveillance, follow-up intensity, and consideration of adjuvant treatment or clinical trial enrollment in high-risk patients, while avoiding unnecessary over-monitoring in patients with lower predicted risk. These results support the potential of this integrated nomogram as a reliable tool for prognostic prediction and clinical decision-making in ccRCC.

### Gene functional enrichment analysis of DEGs

**Figure 3:**
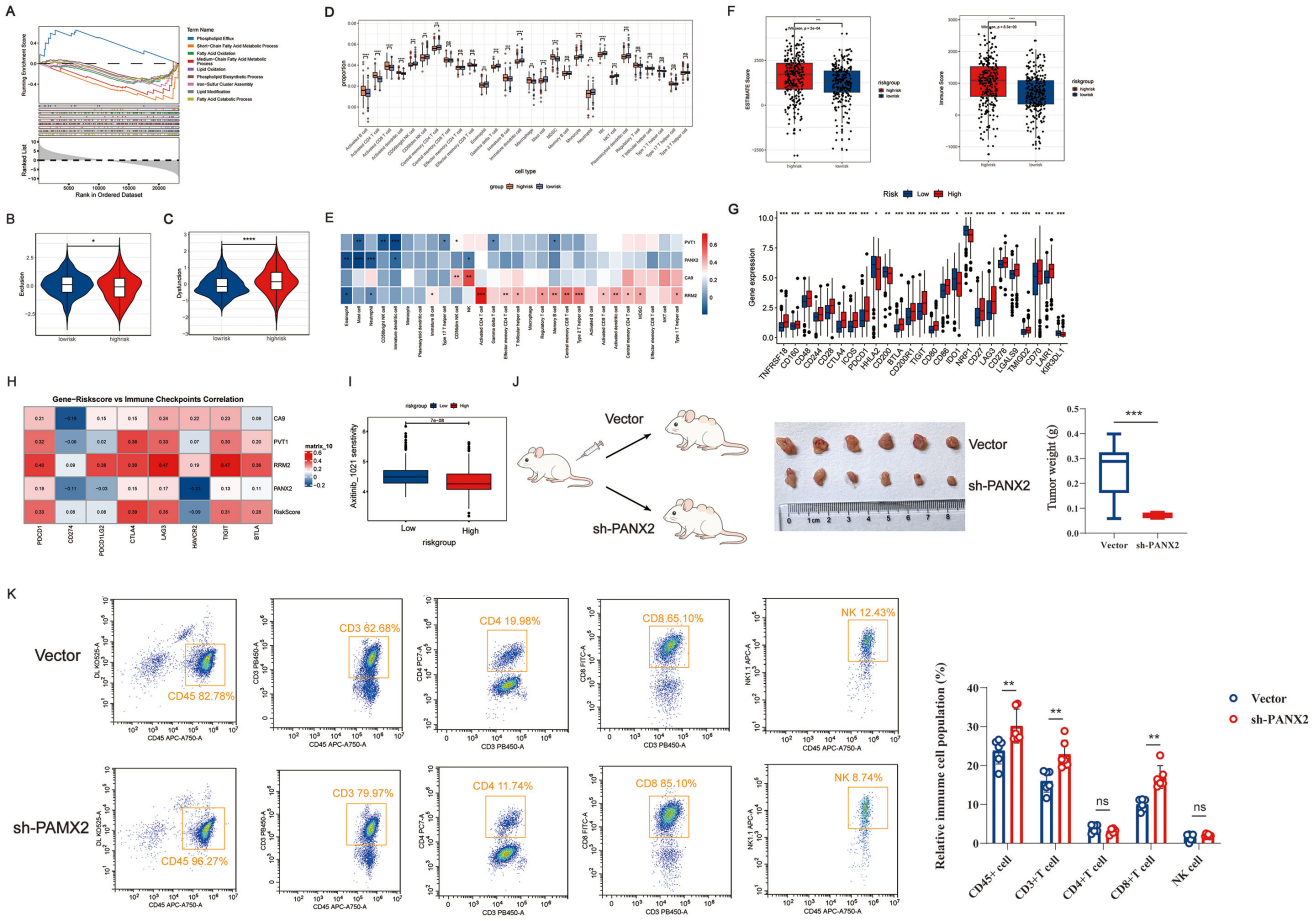
Pathway enrichment, immune landscape, and therapeutic response analysis based on the ferroptosis-related risk model. **(A)** Gene Set Enrichment Analysis (GSA) plot displaying signaling pathways enriched in high-risk and low-risk groups. **(B, C)** ESTIMATE algorithm-derived stromal and immune scores across risk groups. Group differences were evaluated using two-sided Wilcoxon rank-sum tests. **(D)** ssGSEA results illustrating the relative infiltration levels of various immune cell subsets in high-risk and low-risk groups. Group differences were evaluated using two-sided Wilcoxon with B-H correction. **(E)** Correlation heatmap between hub gene expression and myeloid-derived suppressor cell (MDSC) infiltration levels. Correlation coefficients were computed using Spearman’s method (two-sided). **(F)** Analysis of immune dysfunction and exclusion scores between risk groups. Statistical significance was assessed using two-sided Wilcoxon rank-sum tests. **(G, H)** Heatmap and comparative analysis of immune checkpoint gene expression in high- versus low-risk patients. Differences were evaluated using two-sided Wilcoxon tests with false-discovery-rate (FDR) adjustment. **(I)** Drug sensitivity profiling for selected TKIs across risk categories. Group differences were evaluated using two-sided Wilcoxon with B-H correction. **(J)** Tumor weight in immunocompetent BALB/c mice bearing Renca cell grafts at 3–4 weeks post-implantation. **(K)** Flow cytometric analysis of tumor-infiltrating immune cells in immunocompetent mouse models. Data are presented as mean ± SD from at least three independent experiments. Statistical significance was determined as indicated: *p < 0.05, **p < 0.01, ***p < 0.001, ns., no significance, by unpaired *t*-test or one-way ANOVA test.

The signaling pathways in the high-risk and low-risk groups were subjected to Gene Set Enrichment Analysis (GSEA). The result showed that the high-risk group was related to Phospholipid Efflux, suggesting altered phospholipid transport and membrane lipid remodeling. Since ferroptosis is closely linked to the peroxidation of membrane phospholipids, this enrichment may reflect ferroptosis-related lipid metabolic adaptation in high-risk ccRCC. In contrast, metabolic pathways such as short-chain fatty acid metabolism, fatty acid oxidation, medium-chain fatty acid metabolism, lipid oxidation, phospholipid biosynthesis, iron-sulfur cluster assembly, lipid modification, and fatty acid catabolism were more enriched in the low-risk group (**Figure 3A**). These pathways indicate a relatively active lipid catabolic and oxidative metabolic state. Since impaired fatty acid oxidation and lipid accumulation are important features of metabolic reprogramming in ccRCC, enrichment of fatty acid oxidation and lipid catabolism in the low-risk group may reflect better preserved mitochondrial metabolic activity and a less aggressive metabolic phenotype. In addition, iron-sulfur cluster assembly was enriched in the low-risk group, suggesting potential differences in mitochondrial function and redox homeostasis between the two risk groups.

### Ferroptosis-related prognostic signature is associated with distinct immune landscapes

To explore the immunological characteristics associated with the ferroptosis-related risk groups, we performed ssGSEA and ESTIMATE analyses. The ESTIMATE score and immune score were significantly higher in the high-risk group (**Figures 3B, C**). Further immune deconvolution using ssGSEA indicated that the low-risk group was enriched for infiltrations of natural killer cells, central memory CD8+ T cells, immature dendritic cells, and NKT cells (P<0.05, **Figure 3D**). This pattern was corroborated by xCell analysis, which similarly showed increased infiltration of various activated T-cell subsets and antigen-presenting cells in the low-risk group (**Supplementary Figure 5**). Analysis of the hub genes revealed that all four were positively correlated with myeloid-derived suppressor cell (MDSC) infiltration, while immature dendritic cells and monocytes were associated with a favorable prognosis (**Figure 3E**).

Given the established roles of CA9 (19), PVT1 (20), and RRM2 (21, 22) in ccRCC pathogenesis and the limited understanding of PANX2, we focused on elucidating its specific impact on the tumor immune microenvironment. Using an immunocompetent mouse model, flow cytometric analysis of tumors derived from PANX2-knockdown cells revealed a significant increase in the infiltration of CD45+ leukocytes, CD3+ T cells, and CD8+ T cells, while CD4+ T cells and NK cells remained unchanged (**Figures 3J, K**). These findings are consistent with the bioinformatic predictions and suggest that PANX2 knockdown may be associated with a more active cytotoxic T-cell infiltrate in an immunocompetent renal carcinoma setting. However, because the Renca/BALB/c model does not fully recapitulate the immune contexture of human ccRCC, these data should be interpreted as supportive rather than definitive evidence of immune remodeling in human ccRCC.

### The prognostic signature correlates with immunotherapy response

We then explored whether the risk signature was associated with immune-evasion features and therapy-related molecular indicators. The high-risk group showed higher T-cell dysfunction and exclusion scores by TIDE analysis (**Figure 3F**), consistent with a more immune-dysfunctional or immune-excluded phenotype. However, because TIDE was developed primarily in melanoma, these results should be considered hypothesis-generating in ccRCC rather than direct evidence of clinical immune checkpoint blockade response. Consistent with this interpretation, multiple immune checkpoint genes, including CTLA4 and LAG3, were more highly expressed in high-risk tumors (**Figures 3G, H**). For advanced ccRCC patients, where tyrosine kinase inhibitor (TKI) and immune checkpoint inhibitor (ICI) combinations are first-line, drug sensitivity analysis indicated that the high-risk group was significantly less sensitive to Axitinib, a common TKI (P<0.001, **Figure 3I**). These findings suggest that the ferroptosis-related risk signature is associated with distinct immune features and therapy-related molecular indicators, while its predictive value for immunotherapy response in ccRCC requires further validation.

### Single-cell and spatial transcriptomic characterization of ferroptosis-related features

**Figure 4:**
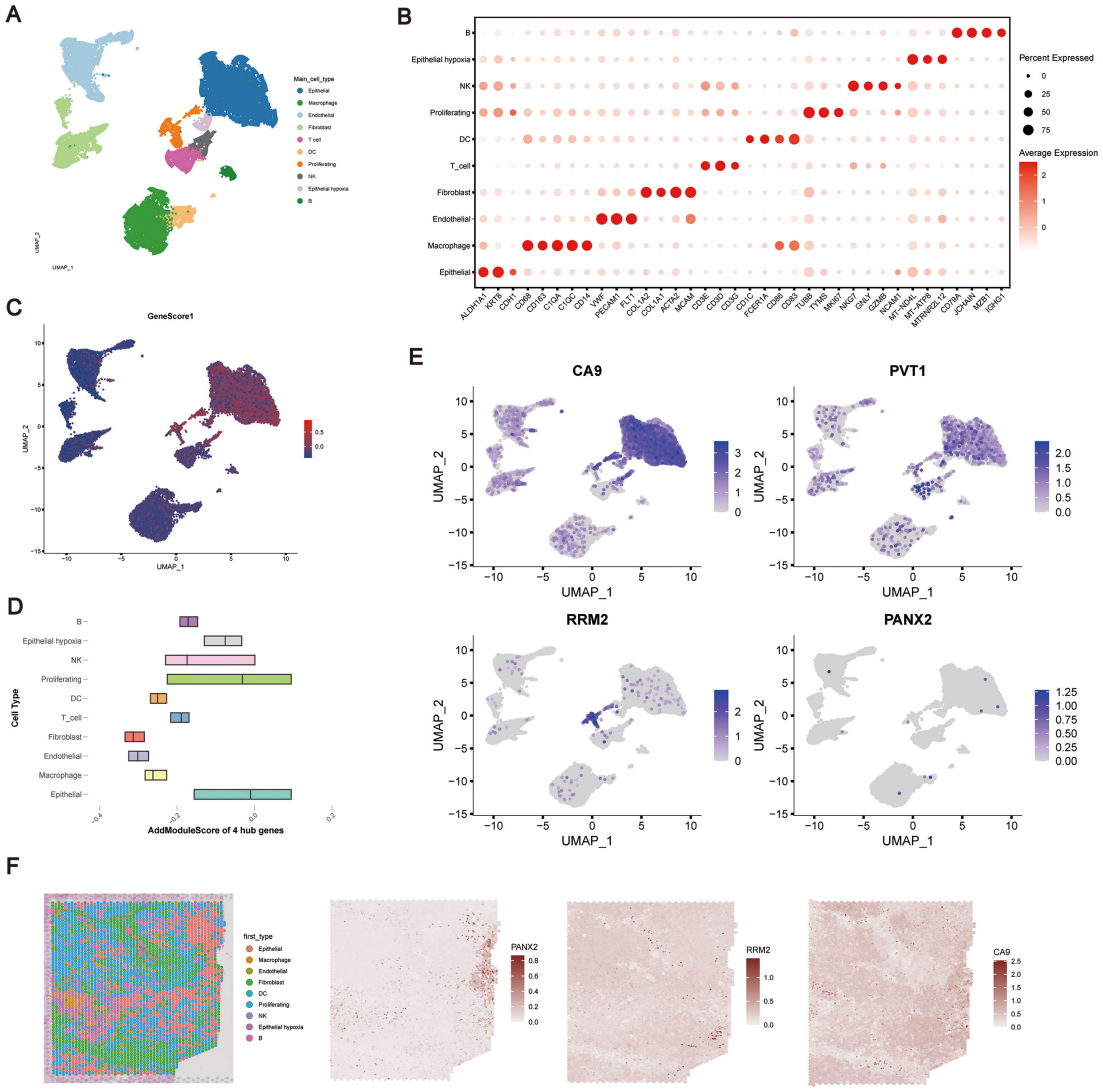
Single-cell and spatial transcriptomic characterization of ferroptosis-related gene expression in ccRCC. **(A)** UMAP projection of 27,478 cells from eight ccRCC samples after quality control and normalization. **(B)** Dot plot showing canonical marker gene expression used for cell type annotation. **(C)** UMAP visualization of ferroptosis-related gene expression score (GeneScore1) across annotated clusters. **(D)** Module scores of four ferroptosis-related hub genes across major cell types. **(E)** Feature plots showing expression of representative hub genes (CA9, PVT1, RRM2, PANX2) in single-cell clusters. **(F)** Spatial transcriptomic mapping (10X Visium) showing cell type distribution (left) and spatial expression of hub genes (PANX2, RRM2, CA9) in ccRCC tissue sections (Spatial transcriptomics data were generated using 10x Visium; each spot represents an area of approximately 55μm in diameter).

To define the cellular context of ferroptosis-related biology, we analyzed scRNA-seq data from eight ccRCC tumor samples in GSE159115. After quality control, 27,478 cells were retained. Unsupervised clustering identified 15 cell populations, which were annotated using canonical markers as B cells, T/NK cells, myeloid cells, stromal cells, endothelial cells, fibroblasts, proliferating cells, and epithelial tumor cells (**Figures 4A, B**).

Ferroptosis-related module scoring showed higher activity in epithelial cells, proliferating cells, and NK cells (**Figures 4C, D**), suggesting that ferroptosis-related programs are not restricted to malignant epithelial compartments but may also intersect with immune-cell states. At single-cell resolution, PANX2 was predominantly expressed in epithelial tumor cells (**Figure 4E**), supporting a primarily tumor-cell-intrinsic role for PANX2 in ccRCC.

We next analyzed the Visium spatial transcriptomics dataset GSE250163 using the integrated scRNA-seq dataset as a reference for spot deconvolution. In this study, spatial transcriptomic analysis served as a complementary layer to the single-cell RNA-seq results. While scRNA-seq identified epithelial tumor cells as the predominant cellular source of PANX2 expression, the Visium data preserved tissue architecture and further demonstrated that PANX2 signals were mainly distributed within epithelial tumor regions across samples (**Figure 4F**). This spatial evidence strengthens the interpretation that PANX2 exerts its major biological effects primarily within the tumor epithelial compartment, providing a spatial context for subsequent functional analyses of PANX2-mediated ferroptosis resistance and tumor progression. Because the current spatial analysis primarily localized PANX2-positive tumor compartments, it should be interpreted as spatial validation of compartmental enrichment rather than as evidence of spatial cell–cell communication.

### Establishment of PANX2-knockdown models and transcriptomic profiling

**Figure 5:**
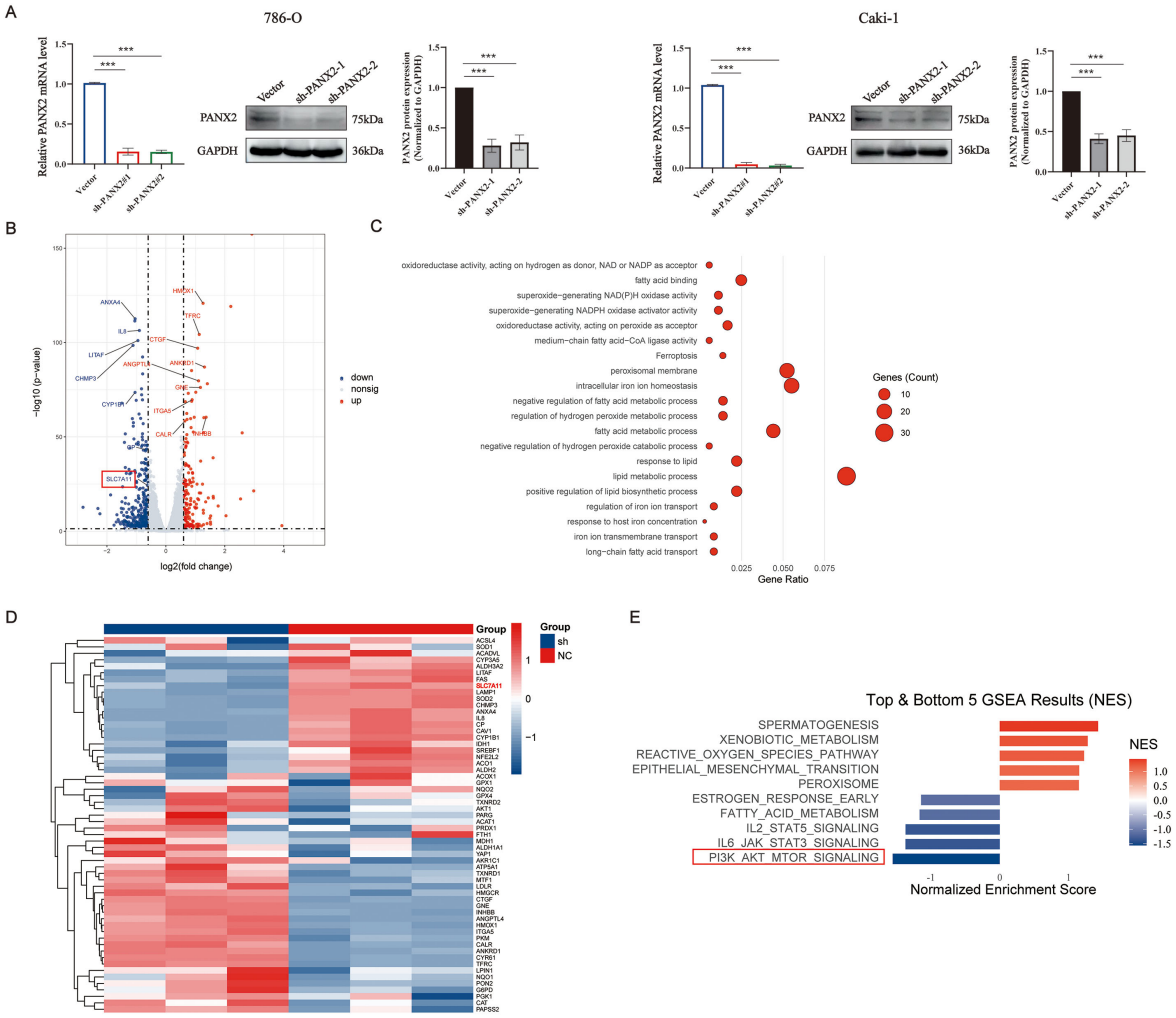
Transcriptomic profiling and pathway analysis following PANX2 knockdown in ccRCC cells. **(A)** Validation of PANX2 knockdown efficiency in 786-O and Caki-1 cell lines via RT-qPCR and Western blot. **(B, C)** Pathway enrichment analysis of differentially expressed genes following PANX2 silencing. **(D)** Heatmap of ferroptosis-related gene expression changes in control versus PANX2-knockdown cells. **(E)** GSEA plot of Akt/mTOR signaling pathway activity. Data are presented as mean ± SD from at least three independent experiments. Statistical significance was determined as indicated: *p < 0.05, **p < 0.01, ***p < 0.001, by unpaired *t*-test or one-way ANOVA test.

To investigate the functional role of PANX2 in ccRCC, we generated stable PANX2-knockdown cell lines (786-O and Caki-1) using lentiviral transduction. Efficient knockdown of PANX2 was validated at both transcriptional and protein levels using RT-qPCR and Western blot (**Figure 5A**). We subsequently performed RNA sequencing to compare the transcriptomic profiles of control and PANX2-knockdown cells. Pathway enrichment analysis revealed that PANX2 silencing was significantly associated with pathways involved in redox homeostasis and ferroptosis (**Figures 5B, C**), suggesting its direct involvement in regulating the metabolic stress responses and ferroptotic cell death in ccRCC.

### PANX2 knockdown impairs antioxidant capacity and elevates lipid peroxidation in ccRCC cells

**Figure 6:**
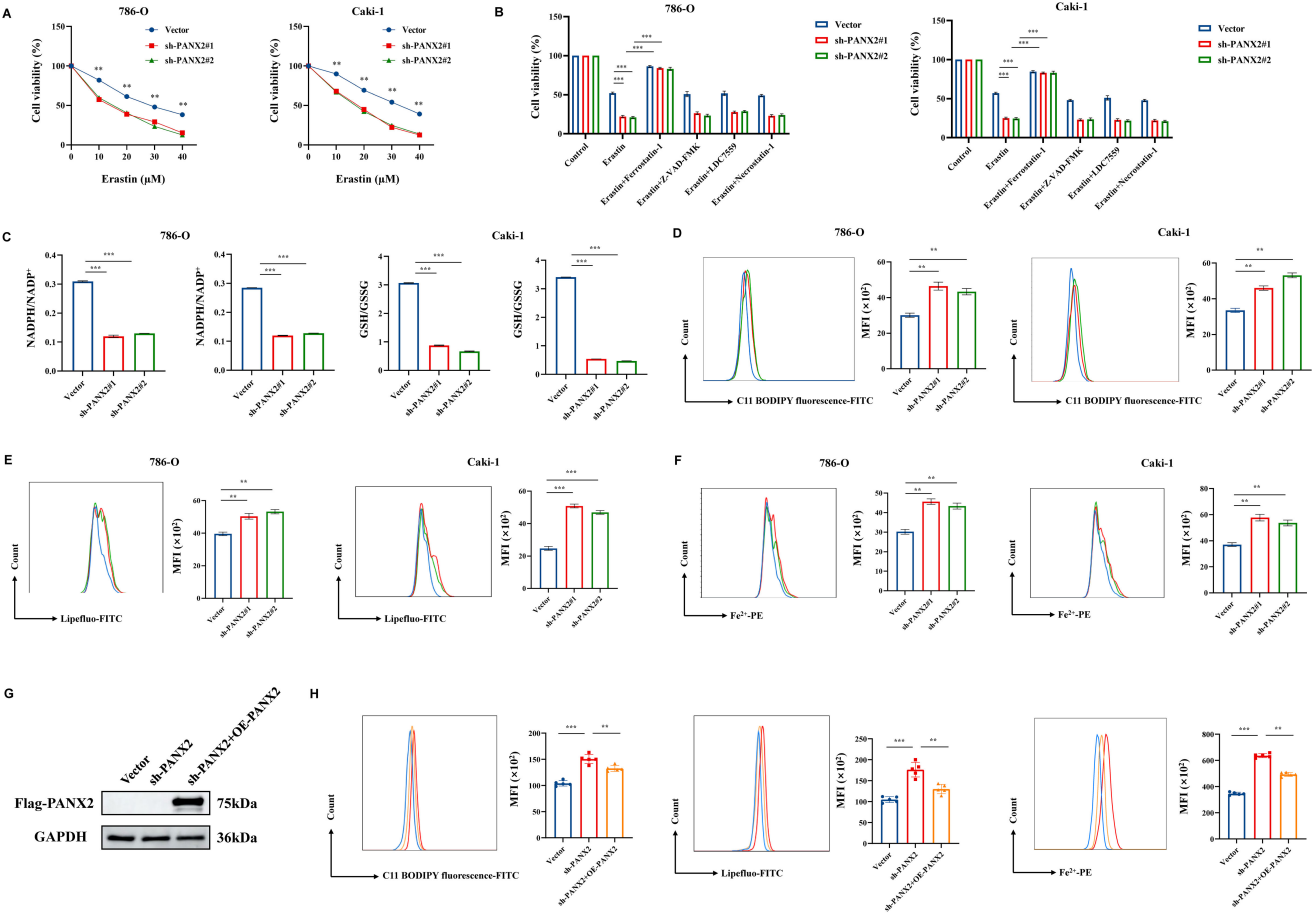
Functional assessment of ferroptosis-related phenotypes upon PANX2 knockdown. **(A)** Dose-response assays for Erastin in sh-PANX2 ccRCC cells. **(B)** Indicated cells were treated with or without 10 μM Erastin for 24h in the presence of different cell death inhibitors (Fer-1, 10 μM; Z-VADFMK, 10 μM; LDC7559, 5 μM; Nec-1, 20 μM). **(C)** Quantitative analysis of NADPH/NADP^+^ and GSH/GSSG ratios in control and PANX2-knockdown cells. **(D, E)** Assessment of lipid peroxidation levels using BODIPY™ 581/591 C11 and Liperfluo fluorescent probes. **(F)** Measurement of intracellular Fe²^+^ levels. **(G)** Western blot analysis of PANX2 expression in rescue experiment cell lines. **(H)** Analysis of lipid peroxidation and intracellular Fe²^+^ levels across experimental groups. Data are presented as mean ± SD from at least three independent experiments. Statistical significance was determined as indicated: **p < 0.01, ***p < 0.001, by unpaired *t*-test or one-way ANOVA test.

To validate these findings, we assessed the functional impact of PANX2 on ferroptosis-related phenotypes. PANX2 knockdown sensitized ccRCC cells to erastin-induced cell death (**Figure 6A**). This enhanced cytotoxicity was specifically rescued by the ferroptosis inhibitor ferrostatin-1, but not by the apoptosis inhibitor Z-VAD-FMK, the necroptosis inhibitor Necrostatin-1, or the pyroptosis inhibitor LDC7559, confirming ferroptosis as the primary cell death modality (**Figure 6B**).

Mechanistically, we found that PANX2 depletion disrupted cellular redox homeostasis. The NADPH/NADP^+^ and GSH/GSSG ratios, key indicators of redox status (23, 24), were significantly reduced in PANX2-knockdown cells (**Figure 6C**). Consistent with the initiation of ferroptosis (6), PANX2 knockdown markedly elevated lipid peroxidation, as measured by BODIPY™ 581/591 C11 and Liperfluo probes, and increased intracellular Fe²^+^ levels (**Figures 6D–F**).

To further confirm the specificity of the phenotype, we performed a rescue experiment by re-expressing PANX2 in the knockdown cells (**Figure 6G**). Reconstitution of PANX2 expression effectively reversed the ferroptotic state, significantly suppressing the elevated lipid peroxidation and Fe²^+^ levels (**Figure 6H**). Collectively, these results demonstrate that PANX2 is a critical regulator that maintains redox balance and constrains ferroptosis in ccRCC.

### PANX2 regulates ferroptosis resistance via Akt/mTOR signaling and promotes tumor growth

**Figure 7:**
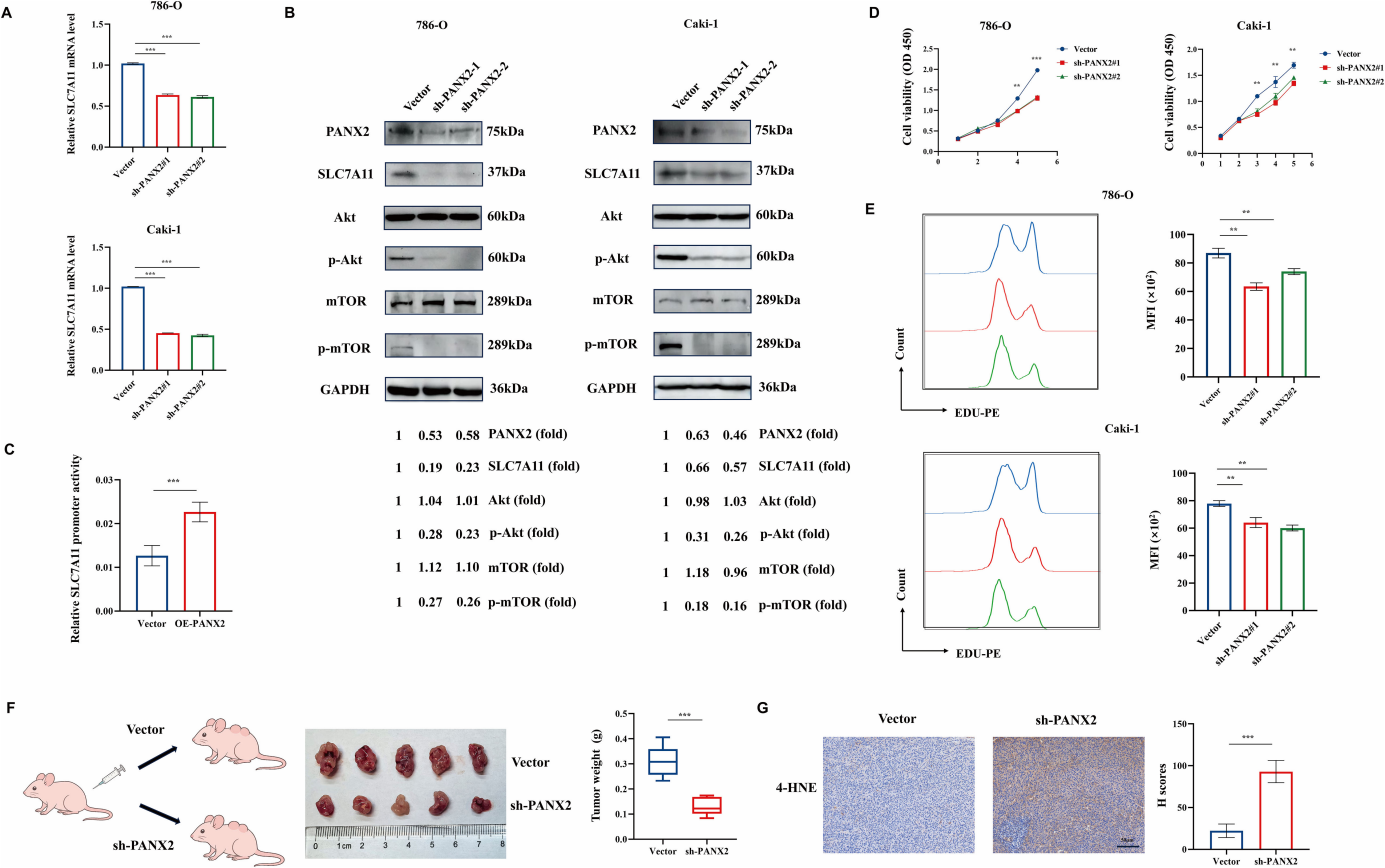
PANX2 regulates the Akt/mTOR/SLC7A11 axis to suppress ferroptosis and tumor growth. **(A)** RT-qPCR analysis of SLC7A11 mRNA expression in control and PANX2-knockdown cells. **(B)** Western blot analysis of SLC7A11, p-Akt, total Akt, p-mTOR, and total mTOR protein levels. **(C)** Luciferase reporter assay for SLC7A11 promoter activity. **(D)** Cell proliferation assessed by CCK-8 assay following PANX2 knockdown. **(E)** EdU incorporation assay evaluating proliferative activity in control and PANX2-deficient cells. **(F)** Xenograft tumor growth analysis in mice implanted with control or PANX2-silenced ccRCC cells. **(G)** IHC staining for 4-HNE in xenograft tumors. Data are presented as mean ± SD from at least three independent experiments. Statistical significance was determined as indicated: **p < 0.01, ***p < 0.001, by unpaired *t*-test or one-way ANOVA test.

Differential expression analysis revealed a marked downregulation of the core ferroptosis regulator SLC7A11 following PANX2 silencing, as visualized by heatmap analysis (**Figure 5D**). This suppression was further confirmed at both the mRNA and protein levels by RT-qPCR and Western blot assays (**Figures 7A, B**). Mechanistically, luciferase reporter assays demonstrated that PANX2 overexpression significantly enhanced SLC7A11 promoter activity (**Figure 7C**), suggesting that PANX2 may influence SLC7A11 transcriptional regulation. However, because PANX2 is a large-pore channel protein rather than a canonical transcription factor, this effect is more likely mediated through upstream signaling events rather than direct promoter binding.

To explore the upstream signaling mechanisms, gene set enrichment analysis (GSEA) revealed that loss of PANX2 was associated with significant suppression of the Akt/mTOR pathway (**Figure 5E**). Consistently, Western blot analysis showed a pronounced reduction in the phosphorylation of Akt and mTOR, whereas total protein levels remained unchanged (**Figure 7B**), confirming pathway inactivation at the signaling level. Given the established role of Akt/mTOR signaling in tumor proliferation (25, 26), we next assessed the functional consequences of PANX2 depletion. PANX2 knockdown significantly impaired cell proliferation as demonstrated by both CCK-8 and EdU incorporation assays (**Figures 7D, E**). These *in vitro* findings were further validated *in vivo*, where PANX2 silencing markedly attenuated tumor growth in xenograft models (**Figure 7F**). Consistent with the induction of ferroptosis, IHC analysis of these tumors revealed a significant increase in 4-HNE staining, a marker of lipid peroxidation, in the sh-PANX2 group (**Figure 7G**).

**Figure 8:**
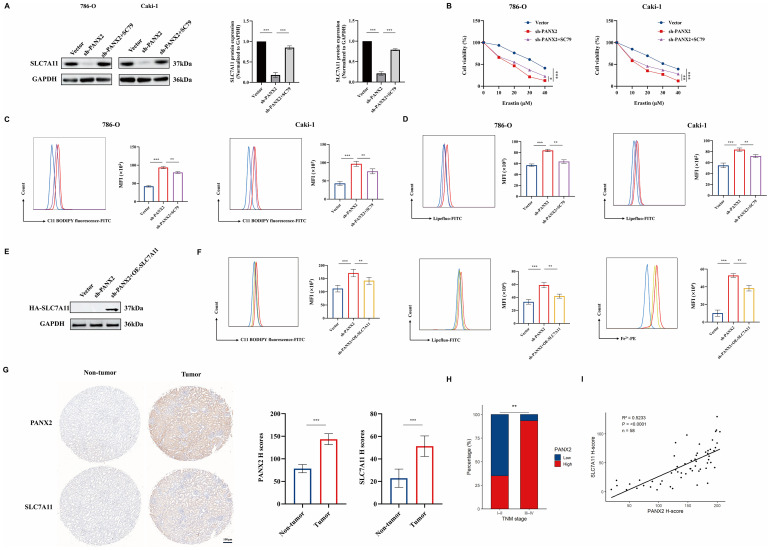
Pharmacological and genetic validation of the PANX2/Akt/SLC7A11 axis, and its clinical relevance in ccRCC. **(A)** Western blot analysis of SLC7A11 expression in PANX2-knockdown cells treated with or without the Akt activator SC79. **(B-D)** Assessment of ferroptosis-related phenotypes following SC79 treatment in PANX2-deficient cells. **(E, F)** Rescue of ferroptosis-related phenotypes by SLC7A11 overexpression in PANX2-knockdown cells. **(G)** Representative IHC images of PANX2 and SLC7A11 in paired tumor and adjacent normal tissues. **(H)** Statistical analysis of PANX2 expression levels across TNM stages I-II versus III-IV. **(I)** Correlation analysis between PANX2 and SLC7A11. Data are presented as mean ± SD from at least three independent experiments. Statistical significance was determined as indicated: *p < 0.05, **p < 0.01, ***p < 0.001, by unpaired *t*-test or one-way ANOVA test.

To directly link Akt/mTOR inactivation to the ferroptosis-sensitizing phenotype, PANX2-deficient cells were treated with the Akt activator SC79. Pharmacological Akt reactivation effectively rescued the enhanced sensitivity to erastin-induced ferroptotic cell death and significantly reduced lipid peroxidation and intracellular ROS accumulation (**Figures 8B–D**). Notably, SC79 treatment also restored SLC7A11 expression in PANX2-knockdown cells (**Figure 8A**). Conversely, ectopic overexpression of SLC7A11 alone was sufficient to reverse the elevated lipid peroxidation and Fe²^+^ accumulation caused by PANX2 depletion (**Figures 8E, F**), supporting SLC7A11 as an important downstream mediator of PANX2-associated ferroptosis resistance.

The clinical relevance of this signaling axis was further evaluated in a tissue microarray comprising 116 ccRCC specimens. IHC analysis revealed coordinated upregulation of PANX2 and SLC7A11 in tumor tissues compared with matched adjacent normal tissues, with a significant positive correlation between their expression levels (**Figures 8G, I**). Importantly, high PANX2 expression was significantly associated with advanced TNM stage (III–IV) (**Figure 8H**), male sex, capsular invasion, and higher Fuhrman nuclear grade, highlighting its strong link to aggressive tumor behavior (**Supplementary Table 6**).

**Figure 9:**
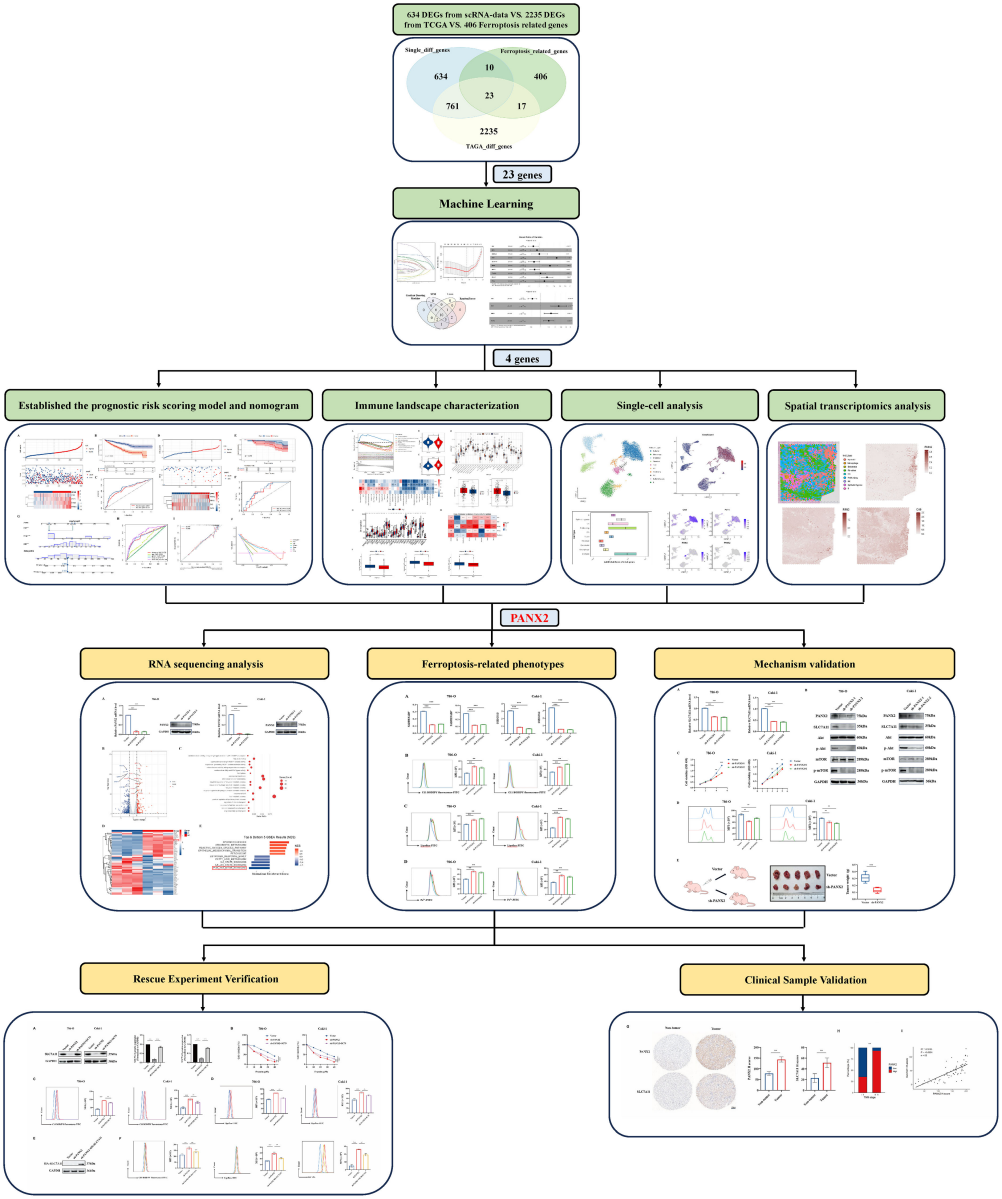
A Flowchart of the study.

Collectively, these results support a model in which PANX2 contributes to ccRCC progression by maintaining Akt/mTOR-SLC7A11-associated ferroptosis resistance. The data identify PANX2 as a clinically relevant biomarker candidate and a potential therapeutic vulnerability, while also highlighting the need for additional mechanistic studies to determine whether PANX2 channel-forming activity, channel-independent scaffold functions, or both are required. To provide a systematic overview of our study, we created a flowchart (**Figure 9**).

## Discussion

This study addresses the need for biologically interpretable prognostic models in ccRCC by integrating scRNA-seq, bulk RNA-seq, spatial transcriptomics, and machine learning to develop a ferroptosis-related prognostic signature. Ferroptosis is particularly relevant to ccRCC because renal cancer cells are metabolically rewired and often lipid-rich, creating both vulnerability to and adaptive resistance against oxidative lipid damage (27, 28). Through this integrative framework, we identified a four-gene prognostic signature and further prioritized PANX2 as a ccRCC-relevant suppressor of ferroptosis. In addition to biomarker discovery, the nomogram integrating molecular risk with clinical variables provides a clinically interpretable framework for individualized risk assessment. By translating the ferroptosis-related signature into patient-level survival probabilities, this model may help clinicians identify patients who require closer surveillance or more intensive therapeutic consideration, while also supporting shared decision-making based on both tumor stage and molecular risk.

The four-gene signature, comprising CA9, PVT1, RRM2, and PANX2, captured both prognostic and biological heterogeneity. Importantly, this four-gene signature should be interpreted primarily as a statistically optimized prognostic model rather than evidence that CA9, PVT1, RRM2, and PANX2 constitute a single linear regulatory pathway. These genes may capture complementary biological dimensions of high-risk ccRCC, including hypoxia/VHL-related metabolic adaptation, lncRNA-mediated transcriptional regulation, nucleotide metabolism and proliferative stress, and PANX2-associated ferroptosis resistance. Thus, the current evidence supports the possibility that these genes operate through partially independent pathways to promote an aggressive tumor phenotype, while direct molecular crosstalk among them remains to be elucidated. The model achieved reproducible and moderate performance, with a C-index of 0.68 in the training cohort and variable time-dependent AUCs across validation datasets. Therefore, its value should not be overstated as a standalone clinical predictor. Rather, its main strength is that it integrates ferroptosis-related biology with prognostic stratification and provides a basis for mechanistic prioritization. The nomogram further suggests that molecular risk may complement age and tumor stage, but prospective validation and comparison with established prognostic systems are required before clinical use. Our analytical strategy aligns with an emerging trend of integrating multi-omics profiling with machine-learning approaches for cell death-related biomarker discovery across different malignancies. A recent study by Gong et al. employed a comparable framework to establish a cuproptosis-related lncRNA signature with prognostic and immunotherapeutic significance in oral squamous cell carcinoma. Although the underlying biological processes differ, these studies collectively highlight the generalizability of integrative computational approaches for identifying clinically informative molecular signatures across cancer types (29).

The pathway analysis provides biological insight into the risk groups. High-risk tumors were associated with phospholipid efflux, which may reflect altered membrane lipid handling and adaptation to lipid peroxidation pressure. In contrast, low-risk tumors showed enrichment of fatty acid oxidation, lipid catabolism, iron-sulfur cluster assembly, and lipid modification pathways, suggesting a more organized metabolic state that may preserve mitochondrial and redox balance. These findings are consistent with the concept that ferroptosis susceptibility is shaped by the coordinated regulation of lipid metabolism, iron handling, and antioxidant capacity rather than by a single marker.

Our most mechanistic finding is that PANX2 supports ferroptosis resistance in ccRCC. Pannexins are large-pore channel proteins involved in intercellular communication and homeostatic signaling (30–32). Previous work implicated PANX2 in colorectal cancer through PI3K-AKT signaling (33), in breast cancer metastasis-associated prognosis (34), and in prostate cancer ferroptosis-associated phenotypes through the Nrf2 pathway (35). Therefore, the novelty of the present work does not lie in linking PANX2 to cancer or ferroptosis in general; rather, it lies in identifying and functionally validating PANX2 as a ferroptosis suppressor in ccRCC and connecting it to a PANX2-Akt/mTOR-SLC7A11-associated mechanism in this disease context. PANX2 knockdown impaired antioxidant capacity, increased lipid peroxidation and Fe2+ accumulation, and sensitized cells to erastin-induced ferroptotic death. Rescue with PANX2, SC79, or SLC7A11 attenuated these phenotypes, supporting a functional axis in which PANX2 helps maintain SLC7A11-dependent redox protection.

The relationship between PANX2 and SLC7A11 should nevertheless be interpreted carefully. Although luciferase reporter assays showed that PANX2 overexpression increased SLC7A11 promoter activity, PANX2 is a large-pore channel protein rather than a canonical transcription factor. Therefore, the observed promoter activation is unlikely to reflect direct transcriptional binding by PANX2. A more plausible explanation is that PANX2 modulates upstream signaling, including Akt/mTOR activity, which may subsequently affect transcriptional regulators or chromatin states involved in SLC7A11 expression. Potential intermediates may include NRF2, ATF4, mTORC1-dependent metabolic transcriptional programs, or other stress-responsive factors that regulate cystine import and antioxidant defense. However, the precise intermediate link connecting PANX2-dependent Akt/mTOR signaling to SLC7A11 transcription remains unresolved. In addition, the present study does not determine whether this regulation depends on PANX2 channel-forming pore activity, channel-independent scaffold functions, or both. Future studies using channel-dead PANX2 mutants, channel-function assays, pharmacological channel blockade, chromatin immunoprecipitation, and SLC7A11 promoter mutagenesis will be required to clarify this mechanism.

The immune analyses suggest that PANX2-mediated ferroptosis resistance may intersect with the tumor immune microenvironment. Bioinformatic deconvolution indicated that low-risk tumors were enriched for NK cells, central memory CD8+ T cells, NKT cells, and antigen-presenting populations, whereas high-risk tumors showed features of immune infiltration coupled with MDSC-associated suppression. In the Renca/BALB/c model, PANX2 knockdown increased CD45+, CD3+, and CD8+ immune-cell infiltration. These observations are consistent with the concept that ferroptotic stress can influence antitumor immunity by altering lipid peroxidation products, damage-associated molecular patterns, antigen presentation, cytokine release, and myeloid-cell recruitment. However, our immune validation used a murine renal carcinoma model rather than human ccRCC tumors, and the human tumor-growth experiments used immunodeficient xenografts. The immune findings should therefore be viewed as supportive and hypothesis-generating rather than definitive evidence for immune remodeling in human ccRCC.

In addition, TIDE analysis and immune checkpoint gene expression suggested that high-risk tumors may be more prone to T-cell dysfunction and exclusion, but TIDE was primarily trained in melanoma and has uncertain predictive accuracy in ccRCC. Accordingly, these results are best considered hypothesis-generating. Future validation should use ccRCC-specific immunotherapy cohorts with matched transcriptomic and clinical response data. If confirmed, targeting PANX2 expression or function could potentially sensitize tumor cells to ferroptosis while reshaping the immune microenvironment, providing a rationale for combination strategies with TKIs or immune checkpoint inhibitors.

Several limitations should be acknowledged. First, although the prognostic model was externally validated, cohort sizes and event numbers were limited, and the GSE167573 time-dependent AUCs showed variability that may reflect cohort-specific sampling effects. Second, the four signature genes were selected for shared ferroptosis annotation and prognostic contribution, but direct biological co-regulation among CA9, PVT1, RRM2, and PANX2 was not established. Third, although validated in our clinical cohort, prospective investigation in larger, multicenter cohorts with more complete clinicopathological variables is warranted to confirm generalizability and to further evaluate its clinical utility with established clinical scoring systems such as SSIGN or UISS. Fourth, the immune microenvironment experiments were performed in a Renca/BALB/c model, whereas human ccRCC tumor-growth experiments used 786-O xenografts in nude mice; therefore, conclusions about immune remodeling in human ccRCC remain indirect. Fifth, although our data support a PANX2-Akt/mTOR-SLC7A11-associated axis, the intermediate transcriptional or epigenetic regulators linking Akt/mTOR signaling to SLC7A11 promoter activity remain undefined, and the study cannot distinguish whether this regulation depends on PANX2 channel-forming activity, channel-independent scaffold functions, or both. Finally, the spatial transcriptomics analysis primarily confirmed epithelial enrichment of PANX2 and did not include spatial co-expression, ligand-receptor interaction, or neighborhood enrichment analyses. Addressing these limitations will require prospective multicenter cohorts, ccRCC immunotherapy datasets, structure-function analyses using channel-dead mutants, channel-function assays, and mechanistic studies of SLC7A11 promoter regulation.

## Conclusion

In conclusion, this study integrates single-cell, bulk, spatial, and machine-learning analyses to identify a ferroptosis-related prognostic signature in ccRCC and to prioritize PANX2 for functional validation. We show that PANX2 supports ccRCC cell proliferation and ferroptosis resistance by maintaining redox homeostasis and modulating an Akt/mTOR-SLC7A11-associated pathway. These findings refine the role of PANX2 in a ccRCC-specific context, provide a biologically interpretable prognostic framework, and nominate PANX2 as a potential therapeutic vulnerability for further preclinical and clinical investigation.

## Data Availability

The public datasets analyzed during this study are available in the following repositories: The Cancer Genome Atlas Kidney Renal Clear Cell Carcinoma (TCGA-KIRC) cohort:https://portal.gdc.cancer.gov/; Single-cell RNA-seq data: Gene Expression Omnibus (GEO) under accession number GSE159115; Spatial transcriptomics (Visium) data: GEO under accession number GSE250163; External validation cohorts: International Cancer Genome Consortium (ICGC) data, E-MTAB-1980, and GSE167573. The custom code used for bioinformatics analysis have been deposited in a GitHub repository: https://github.com/XYQ9999/Paper-analysis-code.git. All other data supporting the findings of this study, including uncropped Western blot images are available within the article and its Supplementary Information files or from the corresponding author upon reasonable request.
