# Supplementary material for: Integrative multi-omics identifies PANX2 as a ferroptosis suppressor and potential therapeutic target in clear cell renal cell carcinoma
Source: Front Immunol. 2026 Aug 3;17:1883667. doi: 10.3389/fimmu.2026.1883667 (PMC13478189; doi:10.3389/fimmu.2026.1883667)
Supplement: Supplementary file 1 [file SupplementaryFile1.docx]

**Title**: Integrative multi-omics identifies PANX2 as a potential ferroptosis suppressor and therapeutic target in clear cell renal cell carcinoma

**Supplementary Figure 1**. **(A–B)** Violin plots of QC metrics before and after filtering. **(C–D)** PCA and Harmony embeddings showing dimensionality reduction and batch correction. **(E–F)** QC feature distributions before and after filtering. **(G)** UMAP visualization of integrated single-cell clusters before Harmony. **(H)** Elbow plot of principal components. **(I)** Cluster relationship graph depicting global cell-state connectivity.

**Supplementary Figure 2.**  (A) Random forest analysis showing model error rates (left) and feature importance ranked by MeanDecreaseGini (right). (B) Cross-validation curve identifying 16 variables with the lowest RMSE. (C) Feature importance ranking by gradient boosting machine (XGB).

**Supplementary Figure 3.** (A) Calibration curves for 3-year and 5-year survival in the TCGA-KIRC training cohort. (B) Calibration curves for 3-year and 5-year survival in the ICGC validation cohort. (C) Overview of the external validation cohort (E-MTAB-1980) used for model assessment. (D) Kaplan-Meier survival analysis of high-risk and low-risk groups in the E-MTAB-1980 cohort. (E) 3- and 5-year ROC curves for E-MTAB-1980 cohort. (F) Calibration curves for 3-year and 5-year survival in the E-MTAB-1980 cohort. (G) Overview of the external validation cohort (GSE167573) used for model assessment. (H) Kaplan-Meier survival analysis of high-risk and low-risk groups in the GSE167573 cohort. (I) 3- and 5-year ROC curves for GSE167573 cohort. (J) Calibration curves for 3-year and 5-year survival in the GSE167573 cohort. (K) Stratified survival analyses across clinical subgroups, including early-stage (I–II), advanced-stage (III–IV), male, female, age ≤60, and age >60. In all subgroups, high-risk patients consistently showed significantly worse overall survival, indicating the robustness and clinical applicability of the risk signature.

**Supplementary Figure 4.** Analysis of the correlation between risk categories (high-risk vs. low-risk groups) and clinical outcomes.

**Supplementary Figure 5.** Immune cell infiltration profiles estimated by xCell between high- and low-risk groups. Statistical comparisons were performed using two-sided Wilcoxon rank-sum tests with FDR correction.
