# Supplementary material for: Integrative multi-omics identifies PANX2 as a ferroptosis suppressor and potential therapeutic target in clear cell renal cell carcinoma
Source: Front Immunol. 2026 Aug 3;17:1883667. doi: 10.3389/fimmu.2026.1883667 (PMC13478189; doi:10.3389/fimmu.2026.1883667)
Supplement: Supplementary file 2 [file Table1.docx]

**Supplemental Table 1**. **The target sequences of shRNAs**

| **Gene name** | **Sequence (5’-3’)** |
| --- | --- |
| PANX2#1 | GCAAGAGCAACTTCATCTT |
| PANX2#2 | CTTCGCAGAGGAACCCATTTACT |
