# Supplementary material for: Integrative multi-omics identifies PANX2 as a ferroptosis suppressor and potential therapeutic target in clear cell renal cell carcinoma
Source: Front Immunol. 2026 Aug 3;17:1883667. doi: 10.3389/fimmu.2026.1883667 (PMC13478189; doi:10.3389/fimmu.2026.1883667)
Supplement: Supplementary file 3 [file Table2.docx]

**Supplemental Table 2**. **Primers used in this study**

| **Primer** | **Sequence** |
| --- | --- |
| PANX2 Forward | CCAAGAACTTCGCAGAGGAAC |
| PANX2 Reverse | GGGCAGGAACTTGTGCTCA |
| SLC7A11 Forward | TCTCCAAAGGAGGTTACCTGC |
| SLC7A11 Reverse | AGACTCCCCTCAGTAAAGTGAC |
| GAPDH Forward | ACAACTTTGGTATCGTGGAAGG |
| GAPDH Reverse | GCCATCACGCCACAGTTTC |
