# Supplementary material for: Integrative multi-omics identifies PANX2 as a ferroptosis suppressor and potential therapeutic target in clear cell renal cell carcinoma
Source: Front Immunol. 2026 Aug 3;17:1883667. doi: 10.3389/fimmu.2026.1883667 (PMC13478189; doi:10.3389/fimmu.2026.1883667)
Supplement: Supplementary file 4 [file Table3.docx]

**Supplemental Table 3**. **The antibodies used in this study**

| **Name** | **Host** | **Dilution ratio** | **Company** |
| --- | --- | --- | --- |
| Anti-PANX2 | Rabbit | 1:1000 | Proteintech |
| Anti-SLC7A11 | Rabbit | 1:1000 | Abcam |
| Anti-Akt | Rabbit | 1:1000 | Cell Signaling Technology |
| Anti-Phospho-Akt | Rabbit | 1:1000 | Cell Signaling Technology |
| Anti-mTOR | Rabbit | 1:1000 | Cell Signaling Technology |
| Anti-Phospho-mTOR | Rabbit | 1:1000 | Cell Signaling Technology |
| Anti-HA |  | 1:10000 | Abcam |
| Anti-GAPDH | Mouse | 1:10000 | Abcam |
| Anti-4-Hydroxynonenal Monoclonal (4-HNE) | Mouse | 1:50 | ThermoFisher |
| APC anti-Mouse CD45 Antibody |  |  | Biolegend |
| BV421 anti-Mouse CD3 Antibody |  |  | Biolegend |
| Percp anti-Mouse CD4 Antibody |  |  | Biolegend |
| Alexa Fluor 700 anti-Mouse CD19 Antibody |  |  | Biolegend |
| PE anti-Mouse CD8 Antibody |  |  | Biolegend |
| PE anti-Mouse F4/80 Antibody |  |  | Biolegend |
| FVS 510 D/L staining |  |  | Biolegend |
| BV650 anti-Mouse NK1.1 Antibody |  |  | Biolegend |
