# Supplementary material for: Integrative multi-omics identifies PANX2 as a ferroptosis suppressor and potential therapeutic target in clear cell renal cell carcinoma
Source: Front Immunol. 2026 Aug 3;17:1883667. doi: 10.3389/fimmu.2026.1883667 (PMC13478189; doi:10.3389/fimmu.2026.1883667)
Supplement: Supplementary file 5 [file Table4.docx]

**Supplemental Table 4**. **The PCR primer sequences for the promoter region of SLC7A11**

| **Primer** | **Sequence** |
| --- | --- |
| Forward | CTGTTTTCCTTTACAGGCTTTTGCTAT |
| Reverse | ACCAGTATTTTAAATCACCATCCGAGTG |
